# Potential for Molecular Testing for Group A Streptococcus to Improve Diagnosis and Management in a High-Risk Population: A Prospective Study

**DOI:** 10.1093/ofid/ofz097

**Published:** 2019-02-26

**Authors:** Anna P Ralph, Deborah C Holt, Sharifun Islam, Joshua Osowicki, David E Carroll, Steven Y C Tong, Asha C Bowen

**Affiliations:** 1 Menzies School of Health Research, Charles Darwin University; 2 Division of Medicine, Royal Darwin Hospital, Northern Territory; 3 Tropical Diseases, Murdoch Children’s Research Institute, and Infectious Diseases Unit, Department of General Medicine, Royal Children’s Hospital, Melbourne; 4 Department of Paediatrics, University of Melbourne; 5 Victorian Infectious Disease Service, Royal Melbourne Hospital, and Doherty Department, University of Melbourne, Peter Doherty Institute for Infection and Immunity, Victoria; 6 Department of Paediatric Infectious Diseases, Perth Children’s Hospital; 7 Wesfarmers Centre for Vaccines and Infectious Diseases, Telethon Kids Institute, University of Western Australia, Perth

**Keywords:** Group A *Streptococcus*, molecular test, point-of-care test, pharyngotonsillitis, rheumatic fever

## Abstract

**Background:**

In high-burden settings, guidelines recommend antibiotic treatment for all suspected group A *Streptococcus* (GAS) infections to prevent rheumatic fever and poststreptococcal glomerulonephritis. Highly sensitive rapid GAS tests could reduce unnecessary antibiotic use in these settings.

**Methods:**

This was a prospective study of the Xpert Xpress Strep A (Cepheid) molecular test compared with culture of throat swab samples collected at a referral hospital in northern Australia. Demographic and clinical data and results of streptococcal serology and culture were collected.

**Results:**

Of 164 throat swab samples, 145 (88%) were eligible for inclusion; 49 (34%) were molecular test positive and 24 (17%) were culture positive for GAS. The sensitivity, specificity, and positive and negative predictive values for the molecular test versus culture were 100.0%, 79.3%, 48.8%, and 100.0%, respectively. Among 25 samples testing positive with the molecular test and negative with culture, group C or G streptococci were cultured in 2, and a plausible clinical explanation, such as pharyngotonsillitis, or rheumatic fever with positive results of streptococcal serology, was apparent in 19 instances. In 25 patients with rheumatic fever or poststreptococcal glomerulonephritis diagnoses, molecular testing nearly trebled the detection of GAS in throat swab samples, from 3 (12%) detected with culture to 8 (32%) detected with molecular testing. Reasons for “false-positive” molecular test results could include the presence of GAS below the threshold of culture detection or persistence of nonviable organisms after infection.

**Conclusion:**

Implementation of molecular testing could improve antibiotic use in this high-burden setting. The incremental yield in poststreptococcal syndromes, by which time cultures are negative, has high potential in the diagnostic workup of autoimmune poststreptococcal syndromes and warrants further investigation.

Infections caused by group A *Streptococcus* (GAS) are an important cause of disease burden globally [1]. GAS infections are prevalent in settings of social disadvantage characterized by household crowding and low access to sanitation and healthcare [2, 3]. For example, in aboriginal communities of northern Australia, many children experience multiple episodes of GAS infection early in life [4], attributable to a range of GAS *emm* types transmitted within households and communities [5, 6]. Repeated infections may prime individuals for autoimmune sequelae, namely, acute rheumatic fever (ARF) leading to rheumatic heart disease and acute poststreptococcal glomerulonephritis (APSGN) contributing to chronic kidney disease. Although there is strong circumstantial evidence that skin GAS infections are implicated in ARF causation [4, 7, 8], the relative proportions of ARF cases in northern Australia triggered by throat or skin infections are unknown.

In northern Australia, treatment guidelines recommend empiric antibiotics for all clinical syndromes consistent with potential GAS infection, such as pharyngotonsillitis or impetigo [9]. Universal antibiotic treatment is recommended because clinical diagnosis is challenging, culture results are inaccessible (owing to remoteness from laboratories), and consequences of missed treatment opportunities are high (eg, ARF). Difficulty distinguishing bacterial from viral pharyngitis may be more pronounced in high-burden settings, owing to a hypothesized immunizing effect of multiple exposures causing an attenuated clinical phenotype [4, 10]. These treatment recommendations contrast with guidelines from low-endemic settings [11], which discourage antibiotic use unless bacterial infection is highly suspected or confirmed. Conventional diagnostic modalities comprise culture and serology; the latter has well-recognized limitations [12, 13]. GAS rapid tests, including point-of-care tests, have been discussed as an option to address diagnostic challenges as part of the Australian national rheumatic fever strategy [14]; GAS rapid tests include rapid antigen detection tests (RADTs) and molecular tests.

RADTs have been used widely in some lower-incidence settings, with demonstrated effectiveness in reducing unnecessary antibiotic prescribing [15, 16]. None are in routine clinical use in Australian aboriginal communities. The sensitivity of RADTs for diagnosis of acute GAS pharyngitis, compared with culture, ranges from 86% to 91% [17, 18]. Performance may be better in adults than in children [18]. Suboptimal sensitivity is the reason RADTs are not recommended for diagnosis of GAS pharyngitis where ARF rates are high [19].

A new generation of molecular GAS rapid tests is now available with higher sensitivity than RADTs [17, 20]. The Xpert Xpress Strep A test (Cepheid) is a molecular test that provides a result in approximately 25 minutes, requiring a desktop laboratory-based analyzer and computer. Although not a true point-of-care test, it is considerably faster than culture, which takes up to 48 hours from the time of receipt in a microbiology laboratory. The assay is an automated real-time polymerase chain reaction (PCR) targeting the *Streptococcus pyogenes speB* gene that encodes streptococcal pyrogenic exotoxin B, a virulence factor universally present in GAS [21]. 

Although *speB* is considered largely specific to GAS, there are reports of expression by non-A streptococci; in 1 study, expression was identified in 3 of 20 group G and C streptococci tested [22], but that study seems to be an outlier, with other research finding that *speB* is unique to GAS [23]. Horizontal transfer of genetic material between β-hemolytic streptococci is a recognized phenomenon [24]. Product information for the Xpert Xpress Strep A test reports a sensitivity, specificity, positive predictive value (PPV), and negative predictive value (NPV) of 100%, 94.1%, 84.1%, and 100%, respectively, based on application to 577 samples in the United States [25] (and personal communication, Rayden Rivett, Cepheid, Australia). Further validation in southern hemisphere indigenous populations is needed.

The aim of the current study was to evaluate the performance of a rapid molecular test compared with bacterial culture of throat swab samples collected at a hospital in tropical northern Australia. Additional aims were to determine test performance according to age, ethnicity, clinical indication for testing (including poststreptococcal syndromes), and results of streptococcal serology. To further evaluate specificity, we also tested pure isolates of non-A β-hemolytic streptococcal human pathogens (groups C and G) using the molecular test platform.

## METHODS

### Setting and Patients

This prospective study was undertaken at Menzies School of Health Research and Royal Darwin Hospital colocated in Darwin, Northern Territory, Australia. Throat swab samples collected for clinical purposes using a cotton-tip swab in Amies gel transport medium and submitted to the hospital’s laboratory for bacterial culture between November 2017 and August 2018 were processed according to routine laboratory methods. Cultures positive for β-hemolytic streptococci were given a semiquantitative grade of 1+, 2+ or 3+. Of these swab samples, any residual sample available in the original transport medium after initial testing in the hospital laboratory was provided to the research laboratory, where patient identifiers were removed and a reidentifiable code applied. The following demographic, clinical, and laboratory data in available electronic medical databases were recorded in an Excel spreadsheet for each sample: patient age, ethnicity, clinical indication for testing, culture result, molecular test result, cycle threshold, and results of serology (antistreptolysin O[ASO] and anti-DNase B [ADB] assays) if available.

Diagnoses were classified as “pharyngotonsillitis” if the clinical diagnosis was tonsillitis, exudative tonsillitis, or pharyngitis, unless “viral” was specified. If the clinical diagnosis was consistent with ARF, these diagnoses were classified as definite, probable, or possible ARF based on documentation by clinicians. Samples were excluded from analyses if the swab was not suitable for culture, if demographic and clinical data were unavailable, or if the anatomic site of collection was not the throat.

### Microbiological and Serological Methods

Throat swab samples received from the hospital laboratory were stored at 4°C for a maximum of 72 hours before processing. Swab sticks were transferred to a modified liquid Amies medium (eSwab; Copan Diagnostics), and 300 µL of the modified liquid Amies medium was loaded into an Xpert Xpress Strep A “research use only” cartridge and analyzed using a GeneXpert IV system, rented from Cepheid. The test provides up to a maximum 43 PCR cycles; samples with lower concentration of DNA require more PCR cycles for detection. An “early assay termination” function provides a positive result when the target DNA signal reaches a predetermined threshold, before completion of all 43 cycles. For the first 50 throat swab samples, those in which GAS was detected with molecular testing were recultured in the research laboratory by plating onto HBA Columbia medium (Thermo Fisher Scientific), incubation for 18 hours at 37°C with 5% carbon dioxide, and observation for the presence of GAS.

To further evaluate specificity, we tested 10 stored isolates each of GAS, group G *Streptococcus* (GGS), and group C *Streptococcus* (GCS). Each set of 10 included isolates recovered from blood, throat carriage, pharyngitis, and skin; each had been previously grouped using an Oxoid Streptococcal Grouping Kit (Thermo Fisher Scientific), and comprised a range of *emm* sequence types [26]. The isolates were cultured as described above, and then 3 colonies were obtained for analysis with an eSwab using the Xpert Xpress Strep A cartridges, as indicated above.

The ASO assays were performed using particle-enhanced immunonephelometry and ADB (Siemens BN II nephelometer). Serological results were interpreted according to age-specific upper limits of normal recommended for use in Australia [12, 27].

### Analyses

Analyses were performed using in Stata IC 14 software (Stata). Figures were produced using Stata or Microsoft Excel (version 14.3.9) software. Contingency tables were used to calculate sensitivity, specificity, PPV, and NPV for molecular testing compared with bacterial culture as the reference standard. Calculations were repeated with stratification by age (<18 or ≥18 years), ethnicity, and major clinical diagnoses, and 95% confidence intervals were calculated using the Clopper-Pearson exact method. Wilcoxon rank sum tests were used for comparisons of nonparametric continuous data, and χ^2^ tests for comparison of categorical data. Regression analysis was used to determine the relationship between cycle threshold for molecular testing and the GAS culture grade. Receiver operating characteristics (ROCs) were examined for cycle thresholds against the reference standard.

### Ethical Approval

This was approved as a low-risk study by the chair of the Human Research Ethics Committee of the Northern Territory Department of Health and Menzies School of Health Research (HREC-2017–2969). The need for additional patient consent was waived because swab samples were collected for clinical purposes and no extra sample collection was undertaken for this research.

## RESULTS

**Figure 1.**
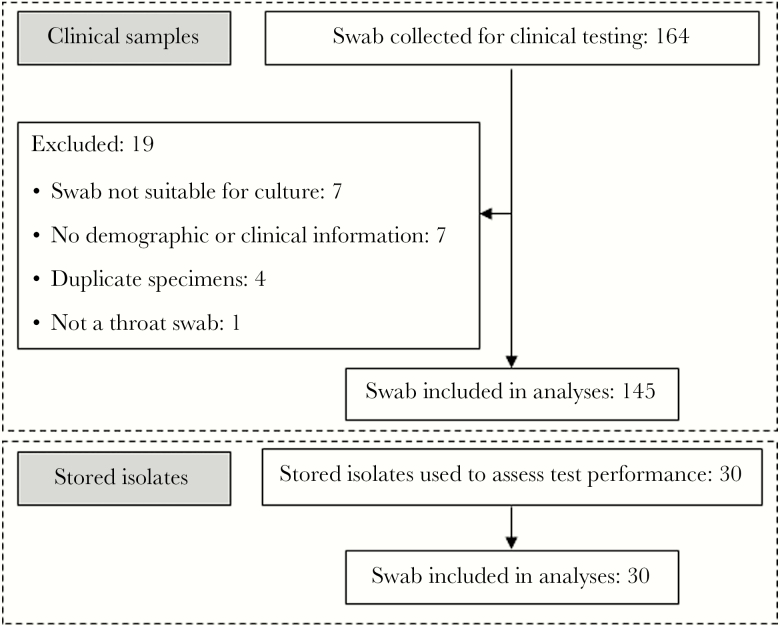
Study flow chart.

**Figure 2.**
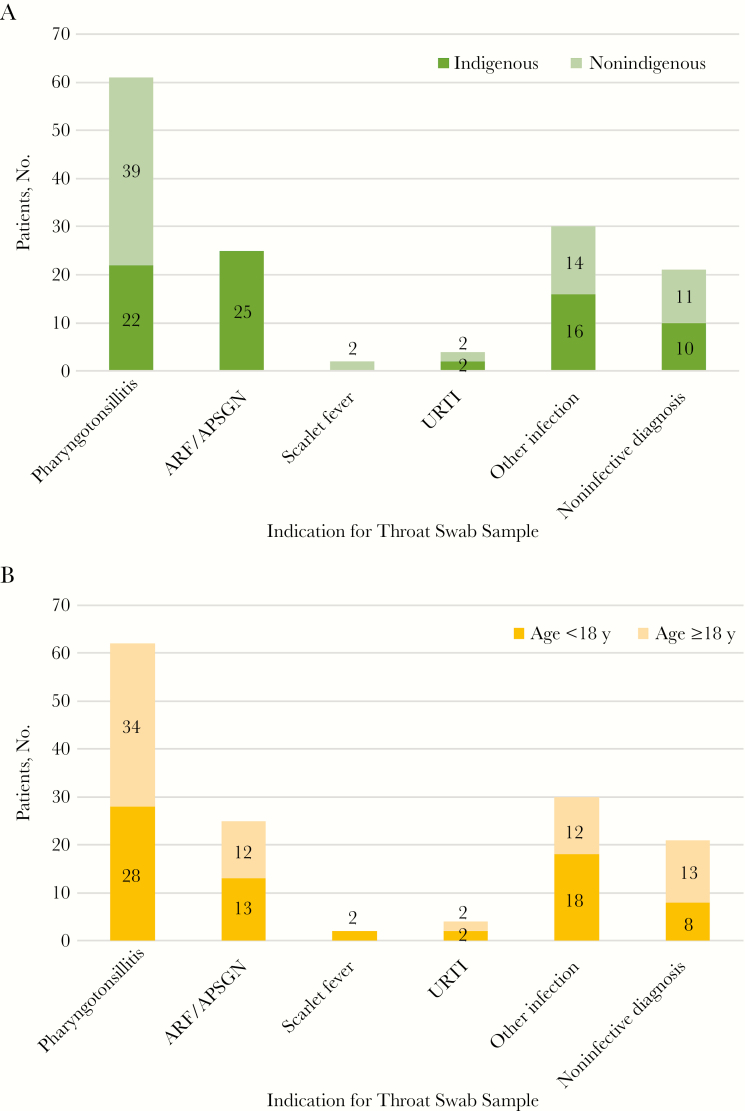
Indications for throat swab samples, according to ethnicity (*A*) and age (*B*). Abbreviations: APSGN, acute poststreptococcal glomerulonephritis; ARF, acute rheumatic fever; URTI, upper respiratory tract infection.

Nineteen of 164 throat swab samples provided to the Menzies research laboratory were excluded (Figure 1) leaving 145 samples, from 145 patients (Table 1). Clinical presentations differed by ethnicity (Figure 2A and Supplementary Table 1). Samples from indigenous persons were more likely to be associated with poststreptococcal autoimmune sequelae (all 25 cases [100.0%] occurred in indigenous persons). A higher proportion of nonindigenous persons presented with symptomatic pharyngotonsillitis (39 of 61 [63.9%]) vs 22 of 61 indigenous persons [36%]; *P* < .001); in 1 pharyngotonsillitis case, the patient’s ethnicity was unknown. Of 25 throat swab samples from persons with poststreptococcal syndromes (definite, probable, or possible ARF and APSGN; Table 1), 8 (32%) were molecular test positive, 3 (12%) were GAS culture positive, and 1 (probable ARF) yielded GCS with negative molecular test results.

**Table 1.** Patient Characteristics, Clinical Indications for Testing and Microbiological Results

Characteristics, Indications, and Results	Patients, No. (%)^a^	
Throat swab samples suitable for analyses, No.	145	
Age, median (IQR), y	17 (9–28)	
Age		
<18 y	73	(50.3
≥18 y	72	(49.7
Indigenous		
Yes	75	(51.7
No	69	(47.6
Not stated	1	(0.7
Clinical indication for testing		
Pharyngotonsillitis	62	(42.8
Definite ARF	10	(6.9
Probable ARF	5	(3.4
Possible ARF	9	(6.2
APSGN	1	(0.7
Scarlet fever	2	(1.4
GAS infection elsewhere	1	(0.7
URTI	4	(2.8
Other infection^b^	31	(21.4
Noninfective diagnosis^c^	20	(13.8
Culture result		
Positive	24	(16.6
Negative	121	(83.4
Molecular test result		
Positive	49	(33.8
Negative	96	(66.2
Results of streptococcal serology^d^		
Positive	35	(24.1)
Negative	22	(15.2)
Not done	88	(60.7)

Abbreviations: APSGN, acute poststreptococcal glomerulonephritis; ARF, acute rheumatic fever; GAS, group A *Streptococcus*; IQR, interquartile range; URTI, upper respiratory tract infection.

^a^Data represent no. (%) of patients unless otherwise specified.

^b^Other infection includes otitis media, gonococcal arthritis, influenza, lower respiratory tract infection, melioidosis, infected wound, impetigo, bacteremia, cellulitis, infected bursitis, infective endocarditis, infective exacerbation of airways disease, scabies, and endometritis.

^c^Noninfective diagnoses include Crohn disease, postviral tenosynovitis, Kawasaki disease, stroke, gout, soft tissue injury, erythema nodosum, and unclear diagnosis.

^d^Streptococcal serology included assays for antistreptolysin O in 57 patients and for anti-DNase B in 47 of the 57.

### Test Performance

Forty-nine samples tested positive with the molecular test, of which 24 were culture positive for GAS, 1 each was positive for GCS and GGS, and 23 were reported culture negative for β-hemolytic streptococci. Swab samples recultured at the research laboratory yielded results that matched those obtained from the clinical laboratory in each instance.

The sensitivity, specificity, PPV, and NPV for the molecular test, compared with culture, were 100%, 79.3%, 48.8%, and 100% respectively (Tables 2 and 3). Subgroup analyses were limited by small numbers, but showed that the PPV was somewhat lower in children (40.0%) than in adults (58.3%), lower in poststreptococcal syndromes (ARF and APSGN) (37.5%) than in pharyngotonsillitis (59.4%), and in accordance with this, lower in indigenous persons (39.1%) than in nonindigenous persons (60.0%) (Supplementary Table 2*A*–2*F*).

**Table 2.** Two-by-Two Paired Contingency Table for Molecular Testing Against Culture

Molecular Test Result	Samples, No.		
	Culture Positive	Culture Negative	Total
Positive	24	25	49
Negative	0	96	96
Total	24	121	145

**Table 3.** Sensitivity, Specificity and Predictive Values for Molecular Test Compared With Culture

Statistical Measure	Result for Molecular Test (95% CI), %
Sensitivity	1.000 (.858–1.000)
Specificity	0.793 (.710–.862)
Positive predictive value	0.489 (.404–.577)
Negative predictive value	1.000 (1.000–1.000)

Abbreviation: CI, confidence interval.

Among the 25 samples that tested positive with the molecular test and negative with culture, a plausible explanation was apparent in most cases (Table 4). Two seemed to be examples of cross-reactivity, with non-A β-hemolytic streptococci isolated by culture. Thirteen were plausible true-positives based on the clinical diagnosis. Six may have represented persistence of GAS DNA after recent infection (5 ARF and 1 scarlet fever case, all with positive serological results). In four cases, the significance of the positive molecular test was uncertain in the given clinical contexts; these may have represented false-positive results (cases 22–25, Table 4).

**Table 4.** Results in 25 Samples With Positive Molecular Testing and Negative Culture Results

Sample	Cycle Threshold, No. of Cycles	β-Hemolytic Streptococci Isolated by Culture	Diagnosis	Positive Results of Streptococcal Serology (ASO or ADB)	Possible Explanation for Positive Results
1	39	GCS (grade 2+)	Monoarthritis	Yes	Cross-reactive with GCS
2	37.5	GGS (grade 3+)	Pharyngotonsillitis	Not done	Cross-reactive with GGS
3	35.1	None	Pharyngotonsillitis	Yes	True-positive
4	32.6	None	Pharyngotonsillitis	Not done	True-positive
5	38.9	None	Pharyngotonsillitis	Not done	True-positive (but note high cycle threshold)
6	36.2	None	Pharyngotonsillitis	Not done	True-positive
7	36	None	Pharyngotonsillitis	Not done	True-positive
8	27.6	None	Pharyngotonsillitis	Not done	True-positive
9	33.9	None	Pharyngotonsillitis	Not done	True-positive
10	38.6	None	Pharyngotonsillitis	Not done	True-positive (but note high cycle threshold)
11	38.1	None	Pharyngotonsillitis	Not done	True-positive (but note high cycle threshold)
12	31.6	None	Pharyngotonsillitis	Not done	True-positive
13	32.5	None	Pharyngotonsillitis	Not done	True-positive
14	31	None	Pharyngotonsillitis	Not done	True-positive
15	31.6	None	Otitis media and “viral” URTI	Not done	True-positive
16	36.5	None	Definite ARF	Yes	Persistent positive after recent infection
17	32.2	None	Definite ARF	Yes	Persistent positive after recent infection
18	34.3	None	Possible ARF	Yes	Persistent positive after recent infection
19	39.2	None	Possible ARF	Yes	Persistent positive after recent infection (but note high cycle threshold)
20	37.2	None	Possible ARF	Yes	Persistent positive after recent infection
21	37.8	None	Scarlet fever	Yes	Persistent positive after recent infection (but note high cycle threshold)
22	42.1	None	Fever after dental extraction; plaque and erythema of soft palate	Not done	Uncertain (note high cycle threshold)
23	32.1	None	Genitolabial abscess	Yes	Uncertain
24	41.8	None	Lower respiratory tract infection	Yes	Uncertain (note high cycle threshold)
25	34.2	None	Melioidosis	No	False-positive

Abbreviations: ADB, anti-DNase B; ARF, acute rheumatic fever; ASO, antistreptolysin O; GCS, group C *Streptococcus*; GGS, group G *Streptococcus*; URTI, respiratory tract infection.

When the stored isolates of GAS, GCS, and GGS were submitted to molecular testing, all 10 GAS isolates were positive, with 1 isolate each of GCS and GGS also positive (Table 5). The molecular test–positive GCS and GGS isolates were emm sequence type (st) stGM220.0 and stC74a.0, respectively.

**Table 5.** Results of Molecular Testing in Stored Pure Isolates

Source of Isolate	Isolates, No.		
	GAS (n = 10)	GCS (n = 10)	GGS (n = 10)
Bacteremia	2	0	2^a^
Abscess	1	1	0
Pharyngitis	2	3	3
Skin sore	2	2	1
Postoperative infection	0	0	1
Asymptomatic (screening)	2	3	2
Throat (symptoms not specified)	1	1^a^	1
Positive for GAS with molecular test	10	1	1
Cycle threshold, no. of cycles	Mean: 16.6	37.3, 39.2 (Repeated)	39.1, 40.2 (Repeated)

^a^Tested positive for GAS with molecular test.

Abbreviation: GAS, group A *Streptococcus*; GCS, group C *Streptococcus*; GGS, group G *Streptococcus*.

### Cycle Thresholds

**Figure 3.**
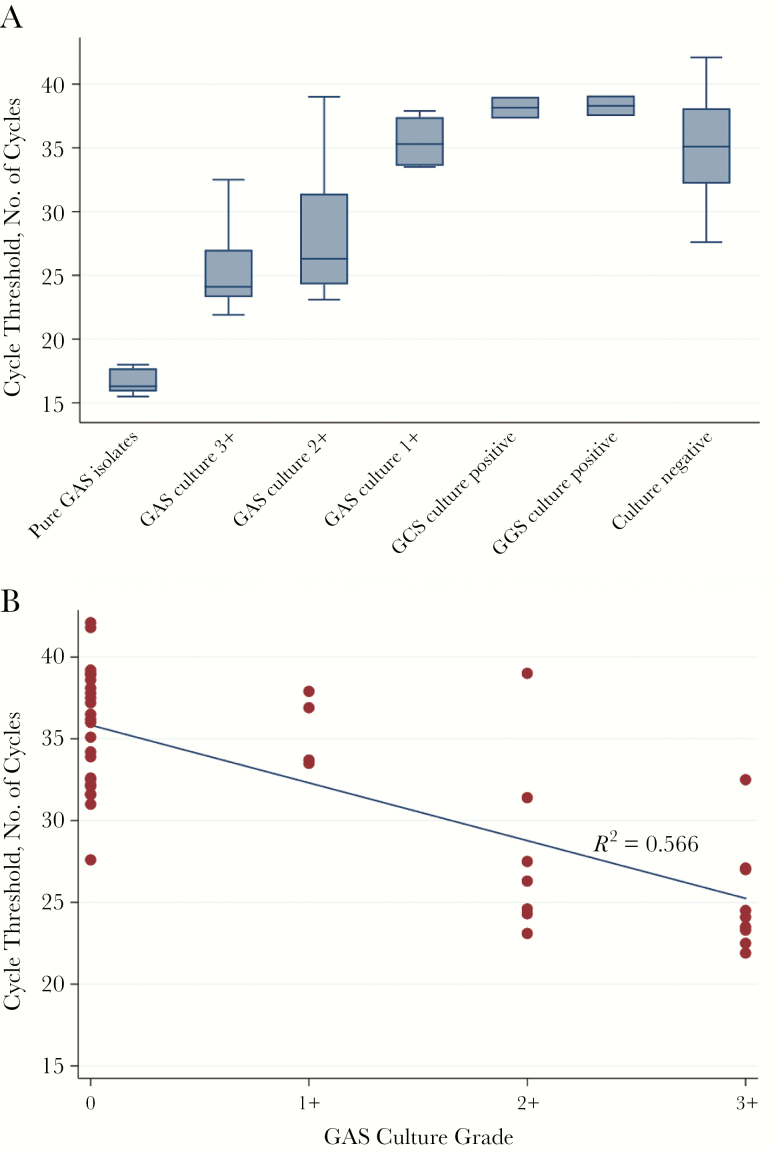
Cycle threshold for molecular test by sample type (*A*) and by grade of group A *Streptococcus* (GAS) (*B*). Culture results were obtained from the hospital laboratory; “culture negative” indicates to no growth of β-hemolytic streptococci. Abbreviations: GCS, group C *Streptococcus*; GGS, group G *Streptococcus*.

Pure GAS isolates were associated with the lowest median number of PCR cycles to detection (16.3 cycles; interquartile range, 15.9–17.7). Cycle time was highest for GCS, GGS, and culture-negative clinical isolates (Figure 3A). Cycle threshold and GAS culture grade were inversely associated (Figure 3B) (*P* < .001; *R*^2^ = 0.566). The median cycle threshold for the 24 GAS culture-positive samples was 26.6 cycles, versus 36.0 cycles for the 25 GAS culture-negative samples (*P* < .001). However, 2 culture-positive samples had high cycle thresholds (37.9 and 39 cycles). The median cycle threshold was slightly lower for samples from patients with a clinical diagnosis of pharyngotonsillitis (31.2 cycles; interquartile range, 24.4–35.9 cycles) than for samples from patients with ARF (35.4 cycles; 32.4–37.6 cycles) (*P* = .07).

For ROC area under the curve analysis, the inverse of cycle threshold was used, with molecular test–negative results allocated a cycle threshold of 44. Using this method, the ROC area under the curve for the cycle threshold as a predictor of positive culture for GAS was highly accurate at 0.95 (Supplementary Figure 1).

### Serological Results

**Figure 4.**
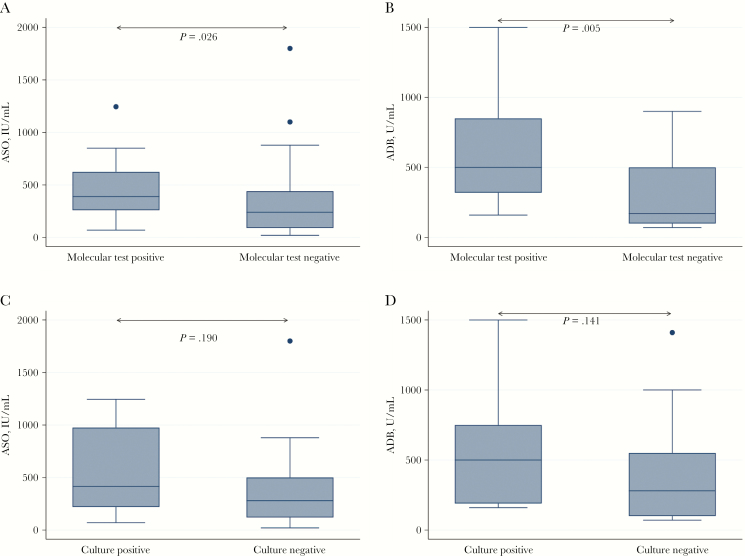
Streptococcal serology results according to molecular testing (A, B) and culture (C, D) Cultures were classified as positive if any β-hemolytic Streptococcus (group A, C, or G) was identified. Abbreviations: ASO, antistreptolysin O; ADB, anti-DNase B.

Results of streptococcal serology for ASO were available for 57 individuals; 47 also had ADB tested. The ASO level exceeded the age-specific upper limit of normal in 34 of 57 individuals (Supplementary Table 3), 13 (38%) of whom had a throat swab sample positive for GAS by molecular testing and 6 (16%) of whom were culture positive for β-hemolytic streptococci (3 GAS, 2 GCS, and 1 GGS). An elevated ASO accompanied ADB elevation (n = 16) except for 1 patient (a 19-year-old with possible ARF) in whom molecular test results and culture were both negative. Median streptococcal serological values were higher in individuals with a molecular test–positive sample than in those with a negative sample (Figure 4) but did not significantly differ according to the culture result.

## DISCUSSION

In a setting affected by high rates of GAS infection and poststreptococcal immune sequelae, the Xpert Xpress Strep A molecular test, compared with culture, had 100% sensitivity for detection of GAS from throat swab samples and detected an additional 19 cases that may have represented true intercurrent or recent infection. There were 6 apparently false-positive molecular test results. The NPV of 100% means that healthcare providers could confidently withhold antibiotics in patients with a sore throat and a negative molecular test result, even in high-risk settings. Advantages of reducing unnecessary exposure to antibiotics include avoidance of antibiotic adverse events and reduced cost. In addition, concerns have been raised in northern Australia that penicillin use may contribute to increasing community rates of methicillin-resistant *Staphylococcus aureus* [28]. However, the true contribution of penicillin to this problem is unclear and other factors may be more important in determining the prevalence of resistant organisms in a community [29].

Two clinical samples and 2 stored isolates that were culture positive only for non–group A β-hemolytic streptococci tested positive with the GAS molecular test. In each instance, the cycle threshold approached the upper limit. Explanations include cross-reactivity with a nucleic acid sequence similar to *speB* or *speB* carriage by GCS/GGS. Non-A streptococcal species do cause pharyngotonsillitis (and skin infection), and hence their detection with a molecular test may still be of value, although their contribution to postinfectious sequelae, including ARF, is uncertain and subject to ongoing investigation [8].

“False-positive” molecular results in clinical samples could be explained by the presence of GAS below the threshold of culture positivity, or detection of DNA from nonviable GAS persisting in the pharynx after acute infection. The latter is suggested by the finding of a stronger association between results of serology and the molecular test than between results of serology and culture (Figure 4). These explanations provide potential reasons for the lower test specificity we identified compared with previous studies of this test [25] or other molecular GAS tests [30]. The duration of positivity of molecular tests after clinical resolution requires better understanding; 1 study found that throat swab samples in most individuals were PCR negative for GAS by 7 days after treatment [31], but the duration of positivity in the absence of treatment is unknown. 

The contribution between asymptomatic “carriage,” associated with lower bacterial load, and false-positive molecular results in the current study is uncertain. There was no opportunity for follow-up serological testing of molecular test–positive, culture-negative, serologically negative individuals, to look for a rise in ASO or ADB levels. These values peak 3–6 weeks and 6–8 weeks after GAS (or GCS/GGS) infections for ASO and ADB, respectively, but the individual and combined sensitivities of these markers re limited [32, 33]. Coincident GAS carriage may occur in viral pharyngitis—in up to an estimated 20% of those with clinical signs of acute pharyngotonsillitis [34]. This has raised concerns that molecular tests could counterintuitively increase the detection of GAS and fail to reduce antibiotic use [35].

There is no single diagnostic test for ARF or APSGN. Diagnosis instead relies on a constellation of clinical and laboratory parameters, including proof of recent streptococcal infection [27]—bearing in mind the limitations of serology noted above. Bacterial cultures of throat swab samples are usually negative by the time the autoimmune symptoms became apparent. If the positive molecular results obtained with throat swab samples from patients with ARF in the current study do represent true-positives, as we are suggesting, then this is a valuable new finding. Of the 25 ARF and APSGN cases, molecular testing nearly trebled the detection of GAS, from 3 (12%) detected with culture to 8 (32%) detected with molecular testing. Restricting analysis to the 10 definite ARF cases, 2 were culture positive and another 2 were positive at molecular testing. The incremental yield of the molecular test was therefore modest but still clinically valuable.

The converse to this is that the majority of individuals with definite, probable, or possible ARF still tested negative for GAS in the throat. The paucity of symptomatic pharyngitis but high frequency of skin GAS infections in our setting have contributed to a revised hypothesis on the pathogenesis of ARF, whereby skin GAS infections lead to throat inoculation to cause ARF, or directly elicit the abnormal immune response that causes ARF [7]. The findings in our study suggest that slightly more patients than previously recognized do have detectable GAS in the throat at the time of ARF diagnosis (by which time serological results have started to rise), but not all of them, and in fact still a minority. In those with negative molecular test results, pharyngeal infection may have occurred in the more distant past, or GAS infection triggering ARF may have not involved the throat. Further study with larger ARF sample sizes are needed to clarify this.

In the current study, symptomatic pharyngitis occurred more commonly in nonindigenous than in indigenous persons, whereas ARF and APSGN were only seen in indigenous persons. This is consistent with previous knowledge that recurrent GAS infections from a young age have 2 outcomes—maladaptive immune priming promoting disproportionate responses, manifested as poststreptococcal autoimmune sequelae, and an “inoculating” effect against symptomatic pharyngitis, attenuating the clinical presentation and making it difficult to distinguish from viral pharyngitis [4]. Scarlet fever was uncommon and was recognized only in nonindigenous individuals.

Limitations of our study include the challenges inherent in evaluating a test wherein the reference standard is less sensitive than the comparator. We considered using a composite reference standard comprising the results of bacterial culture, the molecular test, and the clinical diagnosis of nonviral pharyngotonsillitis, according to recommended approaches [36]. However, clinical diagnosis of bacterial pharyngotonsillitis is so unreliable that this approach proved unhelpful. Available clinical details were scant, and some misclassification may have occurred, because informed consent to scrutinize paper records or interview patients was not obtained. Results of serology were also not available in every instance. However, the level of clinical information collected in our study exceeds that in some other GAS rapid test studies [30].

In conclusion, the current study provides proof-of-concept evidence that rapid molecular testing can inform improved antibiotic use in a high-burden setting. Further investigation is required to explore apparent cross-reactivity with non-A streptococcal species, and the potential to detect nonviable GAS persisting after acute infection in patients with poststreptococcal syndromes. True point-of-care molecular tests could be a feasible and scalable strategy for remote clinics and should be tested in such settings.

## Supplementary Data

Supplementary materials are available at *Open Forum Infectious Diseases* online. Consisting of data provided by the authors to benefit the reader, the posted materials are not copyedited and are the sole responsibility of the authors, so questions or comments should be addressed to the corresponding author.

Supplementary Material
