# Supplementary material for: Potential for Molecular Testing for Group A Streptococcus to Improve Diagnosis and Management in a High-Risk Population: A Prospective Study
Source: Open Forum Infect Dis. 2019 Feb 26;6(4):ofz097. doi: 10.1093/ofid/ofz097 (PMC6469435; doi:10.1093/ofid/ofz097)
Supplement: Supplementary Material [file ofz097_suppl_supplementary_material.pdf]

Supplementary material

Table S1: Characteristics and findings according to clinical syndrome

	Number		Child		Indigenous		Culture positive		Molecular test positive		Serology positive	
	**No.**	**%**	**No.**	**%**	**No.**	**%**	**No.**	**%**	**No.**	**%**	**No.**	**%**
**Pharyngotonsillitis***	62	42.8	28	45.2	22	36.1	19	30.6	32	51.6	3	4.8
**ARF definite**	10	6.9	6	60.0	10	100.0	2	20.0	4	40.0	10	100.0
**ARF possible**	8	5.5	3	37.5	8	100.0	0	0.0	2	25.0	5	62.5
**ARF probable**	5	3.4	3	60.0	5	100.0	1	20.0	1	20.0	3	60.0
**ARF suspected**	1	0.7	0	0.0	1	100.0	0	0.0	1	100.0	1	100.0
**APSGN**	1	0.7	1	100.0	1	100.0	0	0.0	0	0.0	1	100.0
**Scarlet fever**	2	1.4	2	100.0	0	0.0	0	0.0	1	50.0	1	50.0
**GAS infection elsewhere**	1	0.7	0	0.0	0	0.0	0	0.0	0	0.0	0	0.0
**URTI**	4	2.8	2	50.0	2	50.0	0	0.0	0	0.0	1	25.0
**Other infection**	30	20.7	18	60.0	16	53.3	1	3.3	5	16.7	6	20.0
**Non-infective diagnosis**	21	14.5	8	38.1	10	47.6	1	4.8	3	14.3	4	19.0

*ethnicity unknown in one instance

Table S2: Two by two paired contingency tables for Xpert® Xpress Strep A test against culture, according to age category, ethnicity and diagnosis

A. Adults (≥18 years); n=72

		Existing test (Culture)		
		Positive	Negative	Total
**New test (Xpert® Xpress StrepA)**	Positive	14	10	24
	Negative	0	48	48
	Total	14	58	72

		**95% confidence interval**
**Sensitivity**	1.000	0.768 – 1.000
**Specificity**	0.828	0.706 – 0.914
**Positive predictive value**	0.583	0.443 – 0.711
**Negative predictive value**	1.000	1.000 - 1.000

B. Children; n=73

		Existing test (Culture)		
		Positive	Negative	Total
**New test (Xpert® Xpress StrepA)**	Positive	10	15	25
	Negative	0	48	48
	Total	10	63	73

		**95% confidence interval**
**Sensitivity**	1.000	0.858 – 1.000
**Specificity**	0.762	0.638 - 0.860
**Positive predictive value**	0.400	0.300 – 0.509
**Negative predictive value**	1.000	1.000 - 1.000

C. Indigenous; n=75

		Existing test (Culture) Positive	Negative	Total
**New test (Xpert® Xpress StrepA)**	Positive	9	14	23
	Negative	0	52	52
	Total	9	66	75

		**95% confidence interval**
**Sensitivity**	1.000	0.664 – 1.000
**Specificity**	0.788	0.670 – 0.879
**Positive predictive value**	0.391	0.288 – 0.506
**Negative predictive value**	1.000	1.000 - 1.000

D.

D. Non-Indigenous; n=69*

		Existing test (Culture) Positive	Negative	Total
**New test (Xpert® Xpress StrepA)**	Positive	15	10	25
	Negative	0	44	44
	Total	15	54	69

		**95% confidence interval**
**Sensitivity**	1.000	0.782 – 1.000
**Specificity**	0.815	0.686 - 0.908
**Positive predictive value**	0.600	0.462 – 0.724
**Negative predictive value**	1.000	1.000 - 1.000

*Indigenous status missing in one individual

E. Post-streptococcal syndromes (ARF and APSGN); n=25

		Existing test (Culture)		
		Positive	Negative	Total
New test (Xpert® Xpress StrepA)	Positive	3	5	8
	Negative	0	17	17
	Total	3	22	25
			95% confidence interval	
Sensitivity		1.000	0.292 – 1.000	
Specificity		0.773	0.546 - 0.922	
Positive predictive value		0.375	0.217 – 0.565	
Negative predictive value		1.000	1.000 - 1.000	

F. Pharyngotonsillitis; n=62

		Existing test (Culture)		
		Positive	Negative	Total
New test (Xpert® Xpress StrepA)	Positive	19	13	32
	Negative	0	30	30
	Total	19	43	62
			95% confidence interval	
Sensitivity		1.000	0.824 – 1.000	
Specificity		0.698	0.539 – 0.828	
Positive predictive value		0.594	0.481 – 0.697	
Negative predictive value		1.000	1.000 - 1.000	

G.

Table S3: Serological results according to clinical diagnosis

	Antistreptolysin O elevated		AntiDNAse B elevated	
	Number	% out of number tested	Number	% out of number tested
Acute post streptococcal glomerulonephritis	1	100.0	1	100.0
ARF definite	10	100.0	7	87.5
ARF possible	5	55.6	3	37.5
ARF probable	3	60.0	1	25.0
GAS infection elsewhere	0	0.0	0	0.0
Non-infective diagnosis**	4	36.4	1	11.1
Other infection*	6	46.2	3	30.0
Pharyngotonsillitis	3	75.0	1	33.3
Scarlet fever	1	50.0	0	0.0
URTI	1	100.0	0	0.0

ARF: acute rheumatic fever

Definition of elevated serology: see references [12, 27]

*other infection: otitis media, upper respiratory tract infection, gonococcal arthritis, influenza, lower respiratory tract infection, melioidosis, infected wound, impetigo, bacteraemia, cellulitis, infected bursitis, infective endocarditis, infective exacerbation of airways disease, scabies, endometritis

**non-infective diagnoses: Crohn’s disease, post-viral tenosynovitis, Kawasaki disease, stroke, gout, soft tissue injury, erythema nodosum, unclear diagnosis

Figure S1: Receiver-operator characteristics for the cycle threshold* in relation to throat swab culture results (1 = GAS positive, 0 = GAS negative)

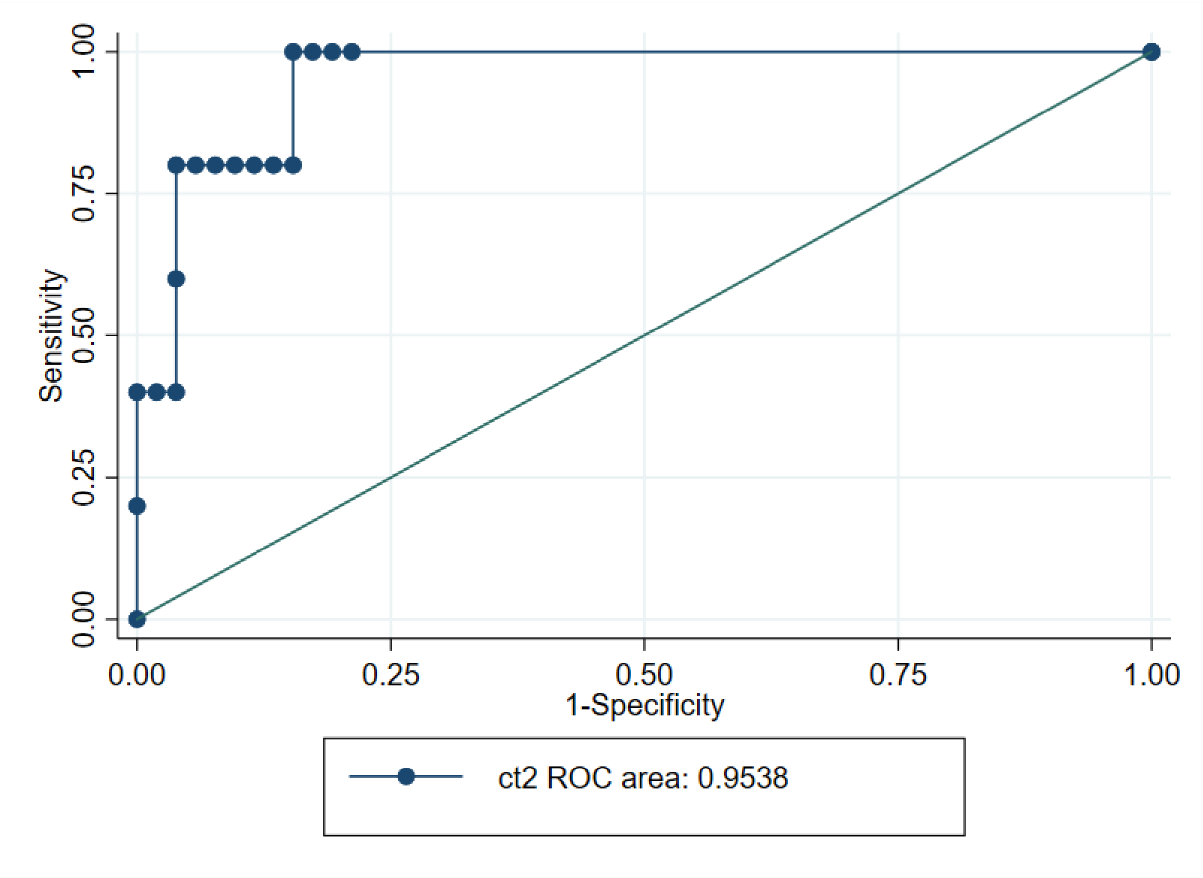

legend: inverse cycle threshold is shown. The cycle threshold value for results which were negative on Xpert® Xpress StrepA testing was given as 44.
